# Melatonin improves muscle injury and differentiation by increasing Pax7 expression

**DOI:** 10.7150/ijbs.79169

**Published:** 2023-01-22

**Authors:** Chen-Ming Su, Chun-Hao Tsai, Hsien-Te Chen, Yi-Syuan Wu, Jun-Way Chang, Shun-Fa Yang, Chih-Hsin Tang

**Affiliations:** 1 Department of Sports Medicine, China Medical University, Taichung City, Taiwan.; 2 Department of Orthopedic Surgery, China Medical University Hospital, Taichung City, Taiwan.; 3 School of Medicine, China Medical University, Taichung City, Taiwan.; 4 Spine Center, China Medical University Hospital, China Medical University, Taichung City, Taiwan.; 5 Program of Biotechnology and Biomedical Industry, China Medical University, Taichung, Taiwan.; 6 Institute of Medicine, Chung Shan Medical University, Taichung, Taiwan.; 7 Department of Medical Research, Chung Shan Medical University Hospital, Taichung, Taiwan.; 8 Department of Pharmacology, School of Medicine, China Medical University, Taichung City, Taiwan.; 9 Chinese Medicine Research Center, China Medical University, Taichung City, Taiwan.; 10 Department of Biotechnology, College of Health Science, Asia University, Taichung City, Taiwan.

**Keywords:** melatonin, Pax7, muscle injury, differentiation, miR-3475-3p

## Abstract

A balance between muscle injury and regeneration is critical for sustaining muscle function during myogenesis. Melatonin is well recognized for its involvement in neuroprotective activities, immune system regulation and suppression of inflammatory responses. This study set out to provide evidence that melatonin improves muscle regeneration during skeletal muscle differentiation. We began with cloning a stable cell line expressing Pax7 knockdown C2C12 cells. We then investigated markers of muscle degradation and regeneration after treating growth medium and differentiated medium with melatonin. Bioinformatics analysis of RNA sequencing results revealed that melatonin regulates muscle differentiation and that Wnt cascades are involved in the mechanism of muscle differentiation. Screening of miRNA online databases revealed that miR-3475-3p is a specific binding site on Pax7 and acts as a negative regulator of Pax7, which is involved in melatonin-induced muscle differentiation. We then investigated the effects of melatonin treatment in the early stage of glycerol-induced skeletal muscle injury in mice. Rotarod performance, micro-computed tomography and immunohistochemistry findings showed that melatonin-induced increases in Pax7 expression rapidly rescue skeletal muscle differentiation and improve muscle fiber morphology in glycerol-induced muscle injury. Our data support the hypothesis that melatonin rapidly rescues skeletal muscle differentiation and the melatonin/Pax7 axis could therefore serve as an important therapeutic target to optimize muscle healing after injury.

## Introduction

Expanding the recovery capacity of skeletal muscle is an important issue. Many physiological and disease processes are contributing risk factors to skeletal muscle health and function 1. During muscle dystrophy, protein markers emerge that are associated with skeletal muscle degradation and wasting, including skeletal muscle atrophy ubiquitin ligase atrogin-1 2, muscle RING-finger-1 (MuRF-1) and MuRF-2 3, and myostatin 4, 5. Specific myogenic markers emerge during muscle regeneration, including myogenic differentiation (MyoD), paired box 7 (Pax7) and insulin-like growth factor 1 (IGF-1) 6, 7. These markers of dystrophy and myogenesis are important for both muscle differentiation and maintaining muscular function.

Skeletal muscle injuries and myopathy contribute to myogenesis and muscle regeneration 8. During myogenesis, myoblasts convert into multinucleated myotubes, which are expressed in various conserved proteins in the cytoplasm, such as myosin heavy-chain (MyHC) 9, desmin 10 and myogenin 11. In muscular atrophy models, the upregulation of Pax7 appears to induce the proliferation of satellite cells that form myocyte sources for future muscle regeneration 12. Previous investigations have indicated that Pax7 acetylation regulates satellite cell self-renewal and the potential for muscle stem cell differentiation 13. The muscle regeneration process is tightly regulated by Pax7 and various structural proteins in the satellite cells 14. However, the recovery strategy of skeletal muscle injuries in myoblasts requires further elucidation.

Cell-based muscle differentiation is characterized as a heterologous or autologous approach that reconstitutes and ameliorates muscle dystrophy 15. The secretion of melatonin from the pineal gland acts as a powerful antioxidant to protect neuron function and promote myocardial differentiation 16, 17. Notably, melatonin partially alleviates the inhibitory effects of hypoxia upon cardio-myocyte differentiation 18, while an inverse relationship has been observed between urinary melatonin and sarcopenia in postmenopausal women, suggesting that melatonin may protect against sarcopenia 19. However, it remains unclear as to whether melatonin plays a role in skeletal muscle dystrophy. We therefore investigated the significance of melatonin-induced increases in Pax7 expression in skeletal muscle myoblasts and differentiated myocytes, and we used muscle injury mouse models in an attempt to define the mechanism by which melatonin-induced signaling improves outcomes after muscle injury, to determine whether this mechanism translates into a potential therapeutic strategy.

## Materials and Methods

### Materials

All the antibodies, inhibitors, small-interfering RNA (siRNA), primers and recombinant proteins were shown in **Supplementary Materials**.

### Cell cultures and stable knockdown cell clones

The murine myoblastic cell line C2C12 was purchased from the American Type Culture Collection (Manassas, VA, USA) and maintained in 5% CO_2_ at 37°C. Cells were cultured in Dulbecco's Modified Eagle's Medium (DMEM) (Gibco, Grand Island, NY, USA) supplemented with 10% fetal bovine serum (Gibco, USA) containing antibiotics (100 U/mL penicillin, 100 µg/mL streptomycin). C2C12 myoblast cells were cultured in differentiated medium (DM) which is DMEM containing 2% horse serum medium for 3 days to induce myotubes 20.

The Pax7-knockdown C2C12 cell line was stably cloned using a lentivirus, according to a previously described method 21. Recombinant lentiviruses were produced in 293T cell lines with a short hairpin RNA-expressing plasmid (TRCN0000365913), packaging plasmid pCMVΔR8.91, and the VSV-G envelope glycoprotein expression plasmid (pMD.G). All plasmids were obtained from the National RNAi Core Facility of the Academia Sinica (Taipei, Taiwan). For generation of stable cell lines, stably-infected Pax7-knockdown C2C12 clones were selected with puromycin (2 mg/ml) for 1 month. Knockdown efficacy of the *Pax7* gene was confirmed by immunoblotting analysis.

### RNA sequencing and data analysis

Total RNA of the C2C12 cells with or without melatonin treatment were isolated for RNA sequencing. The RNA quality and integrity were examined using Bioanalyzer 2100 and RNA 1000 Nano LabChip Kit (Agilent), and the sample with RNA integrity number less than 7 was excluded from the subsequent assay. After mRNA fragmented and cDNA library prepared, the RNA-sequencing was investigated by Illumina HiSeq 4000 (paired-end, 150 base pairs, PE150) and mapped by using HISAT package (http://ccb.jhu.edu/software/hisat2). EdgeR was used to estimate the differentially regulated genes of all transcripts by calculating fragments per kilobase per million (FPKM). The differentially expressed genes were determined with log2 (fold change) >1 or log2 (fold change) <-1 and with statistical significance (p value < 0.05) by R package.

### Quantitative real-time polymerase chain reaction

TRIzol^TM^ reagent was used for extraction of total RNA from murine myoblast cells (MDBio, Taipei, Taiwan). Quantitative real-time polymerase chain reaction (qRT-PCR) analysis was conducted as according to our previous reports 22. All results are calculated by StepOne software version 2.3, and obtained from 6 independent experiments performed in duplicate.

### Immunofluorescence staining

All cells were treated with melatonin (100 ng/ml) at 3 days post-differentiation, then fixed, permeabilized, and labeled with various primary antibodies. Goat anti-rabbit or goat anti-mouse IgG cross-adsorbed secondary antibody Alexa-Fluor^®^ 488 conjugate (Thermo Fisher Scientific, Hemel Hempstead, UK) was applied with a fluorescent microscope (Carl Zeiss, Oberkochen, Germany). 4,6-Diamidino-2-phenylindole (DAPI) was used for staining nuclei. The fusion index was defined as the number of nuclei in myotubes divided by the total number of nuclei present in the observed field.

### Glycerol-induced skeletal muscle injury mouse model

Eight-week-old male C57BL/6J mice were purchased from the National Laboratory Animal Centre (Taipei, Taiwan). The animal use protocol has been reviewed and approved by the Institutional Animal Care and Use Committee at China Medical University (certified numbers: CMUIACUC-2021-139). The mice were randomly separated into three groups: a control group; a skeletal muscle injury group; and a skeletal muscle injury group administered oral melatonin (n=10 for each group). We used glycerol to induce skeletal muscle injury, according to previously described methodology 23. Briefly, intramuscular injections of sterilized glycerol (70 µl of 50%, v/v) were delivered into the tibialis anterior (TA) muscles of both hind limbs at 0 day. Muscle injury was induced within the first 3-6 days. A rotarod device was used to perform muscular endurance, and mice were running on a rotating cylinder at a speed of 40 revolutions per minute (rpm) for 30 min (Singa Technology Corporation, Taiwan). After the mouse fell off from the rotating cylinder, running time was measured and recorded. The rotarod results were measured every 2 days before the mice were sacrificed at 9 days.

### Microcomputed tomography analysis

TA tissue samples were isolated and stained with phosphotungstic acid (PTA) for 30~45 days, followed by using micro-computed tomography (micro-CT) imaging. The scanning protocols of micro-CT used 10 W output, 142 μA current and 70 kVp X-ray voltage, with a 0.5-mm aluminum filter. Image reconstruction was performed using graphics processing unit (GPU)-based reconstruction software, and GPU-Nrecon (Bruker micro-CT, Kontich, Belgium). TA muscle areas were analyzed by using 2 mm images (236 slices). The beam-hardening and ring artifacts were chosen for corrections by using CTAn software (Ver. 1.20.8, Bruker micro-CT, Kontich, Belgium). After the region of interest was selected, reconstructed cross-sections were re-orientated. Murine bones of tibias and femurs were also detected by using an *ex vivo* micro-CT scanner, Bruker Skyscan 1272 (Bruker micro-CT, Kontich, Belgium) at 8.5 μm voxel resolution.

### Immunohistochemistry

The TA muscles were embedded in paraffin, then rehydrated and stained with hematoxylin and eosin (H&E), according to previous reports 24. Immunohistochemistry (IHC) was performed using an IHC Kit (Sigma-Aldrich, St. Louis, MO, USA), according to the manufacturer's instructions.

### Dystrophin staining

Paraffin-embedded sections were prepared and labeled with primary rabbit anti-dystrophin polyclonal antibody (Abcam, Cambridge, UK) antibody at 4°C overnight. Sections were then incubated with goat anti-rabbit IgG cross-adsorbed secondary antibody Alexa-Fluor^®^ 594 conjugate (Thermo Fisher Scientific, Hemel Hempstead, UK). The nuclei were then stained with DAPI. The dystrophin-stained sections were examined with TissueFAXS^®^ Spectra systems (TissueGnostics, Vienna, Austria). A cross-sectional area of dystrophin-positive muscle fibers was quantified using ImageJ software.

### Statistical analysis

Statistical analyzes were performed with Graph Pad Prism software version 8.2.1 (GraphPad Software, La Jolla, CA, USA). Data in all figures are presented as the mean ± standard deviation (SD). Differences between selected pairs from the experimental groups were analyzed for statistical significances using the Student's *t*-test. Statistical comparisons among three or more groups used two-way ANOVA. Between-group differences were considered significant if *p*-values were less than 0.05.

## Results

### Pax7 expression was upregulated during muscle differentiation

**Figure 1:**
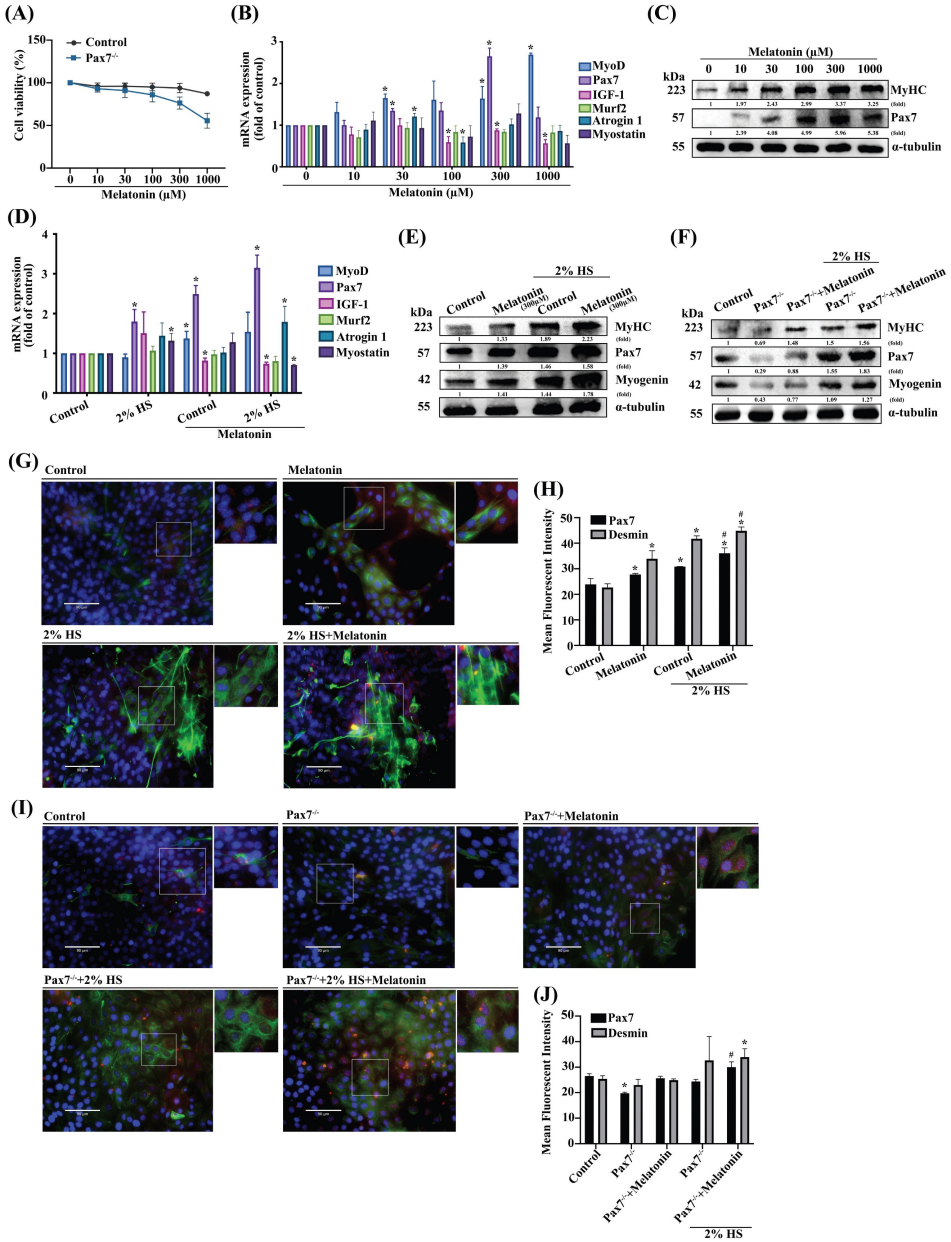
** The role of Pax7 in myoblastic differentiation. (A)** Wild-type (WT) C2C12 cells and Pax7 knockdown C2C12 (Pax7^-/-^) cells were incubated with different concentrations of melatonin for 24 h, and cell viability was examined (N=3). **(B)** mRNA levels of muscle atrophy and myogenic markers in C2C12 cell lines after 24 h of treatment with different concentrations of melatonin (N=3).** (C)** Myogenic markers MyHC and Pax7 were examined after 24 h of treatment with different concentrations of melatonin (N=3). **(D)** WT C2C12 cells were treated with different concentrations of melatonin for 24 h in growth medium and differentiated medium (DM), and the mRNA levels of muscle atrophy and myogenic markers were examined (N=3).** (E)** Levels of protein expression of myogenic markers MyHC, Pax7 and myogenin were examined after 24 h of treatment with different concentrations of melatonin in growth medium and DM (N=3).** (F)** Levels of MyHC, Pax7 and myogenin protein expression in WT and Pax7^-/-^ cells were examined after 24 h of treatment with melatonin in growth medium and DM (N=3). **(G)** WT C2C12 cells were treated with melatonin or left untreated and double-stained with immunofluorescent antibodies to identify Pax7 and desmin in growth medium and DM. Pax7 is depicted by red coloring; desmin by green. Nuclei are shown in blue (N=3).** (I)** WT and Pax7^-/-^ cells were treated with melatonin or left untreated and double-stained with immunofluorescent antibodies to identify Pax7 and desmin in growth medium and DM. Pax7 is depicted by red coloring; desmin by green. Nuclei are shown in blue. **(H & J)** The intensity of immunofluorescent staining was calculated and quantified (N=3). Results are expressed as the means ± SD of at least 3 independent experiments. *p < 0.05 compared with controls or the WT group; #*p* < 0.05 compared with the melatonin treated-group. HS, horse serum.

It is established that most muscle adult satellite cells express Pax7 25, although the role of Pax7 in melatonin-induced regulation of myoblast differentiation is unclear. We therefore cloned a C2C12 cell line stably expressing Pax7 knockdown (Pax7^-/-^) to initially compare the viability of such cells with that of wild-type C2C12 cells **(Figure 1A)**. The efficiency of the Pax7 knockdown was shown as **Supplementary Figure S1**. To examine the role of melatonin in muscle differentiation, muscle regeneration markers were dose-dependently induced after melatonin treatment **(Figure 1B)**. We also measured the protein expression of muscle differentiation markers, including MyHC and Pax7 **(Figure 1C)**. To further examine the effects of melatonin on differentiated C2C12 cells, we treated them with DM to initiate the development of myoblasts into myotubes. We observed increases in some markers of muscle regeneration in DM-treated wild-type C2C12 cells after melatonin treatment **(Figure 1D)**, as well as detectable levels of protein expression for MyHC, Pax7 and myogenin in DM-treated Pax7 knockdown C2C12 cells after melatonin treatment **(Figure 1E-F)**. Immunofluorescent double-staining results revealed the upregulation of Pax7 and desmin in DM-treated wild-type C2C12 cells after melatonin treatment **(Figure 1G)**, as well as in Pax7 knockdown C2C12 cells **(Figure 1I)**; these results are quantified in **Figure 1H and 1J**, respectively. Thus, melatonin appears to regulate myoblast differentiation via the Pax7 marker.

### Bioinformatics analysis confirmed that melatonin regulates muscle differentiation in myoblastic cells

**Figure 2:**
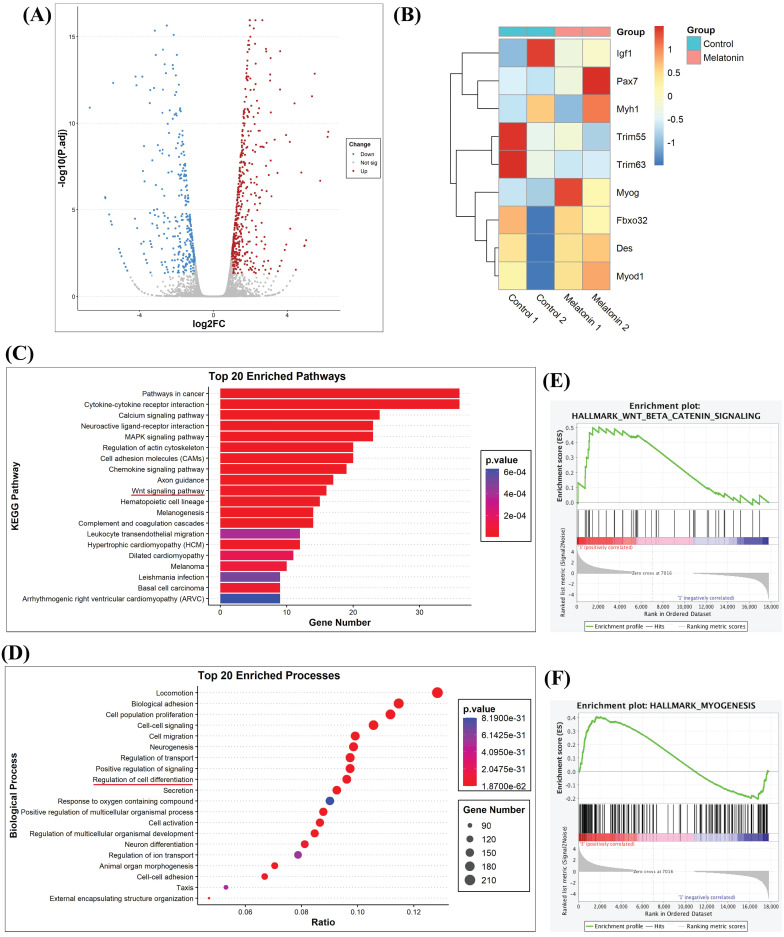
** RNA sequencing results reveal that melatonin regulates gene-regulated skeletal muscle differentiation in C2C12 cells. (A)** Volcano plot of RNA sequencing showing differentially expressed genes in C2C12 cells with or without melatonin treatment. **(B)** The heat map analysis shows differentially regulated skeletal muscle development genes in the control group and the melatonin treatment group; data are presented as log_2_ scale. **(C)** Kyoto encyclopedia of genes and genomes (KEGG) pathways show the normalized enrichment scores of the top 20 enriched pathways. **(D)** Gene Ontology Enrichment analysis categorized the pathways that were significantly altered after melatonin treatment, with highlighting depicting the regulation of cell differentiation. **(E-F)** Gene set enrichment analysis (GSEA) plot presenting melatonin treatment in association with Wnt/β-catenin signaling **(E)** and hallmark myogenesis **(F)**.

The antioxidant activity of melatonin protects against muscle atrophy in inflammatory diseases 26. In order to determine potential targets of melatonin that may regulate the biological process of myoblastic differentiation, RNA sequencing analyzed myoblastic cells treated with or without melatonin. Subsequent volcano plot analysis revealed a total of 3,470 genes with significant levels of expression; 1,795 genes were upregulated and 1,675 were downregulated in melatonin-treated cells compared with untreated control cells **(Figure 2A)**. To identify functional genes associated with skeletal muscle differentiation, we listed 8 representative genes in a heat map. As shown in **Figure 2B**, the expression levels of *Pax7* and* myog* (myogenin) were increased, while the other myogenic genes were decreased compared with untreated controls **(Figure 2B)**. When differentially expressed genes identified in different myoblast groups were subjected to Kyoto Encyclopedia of Genes and Genomes (KEGG) pathway analysis, this revealed 1,362 differentially expressed genes annotated into 89 pathways that included cancer, calcium, regulation of cytoskeleton activity, and Wnt signaling **(Figure 2C)**. Next, we performed Gene Ontology (GO) enrichment analysis, which revealed enrichment of many biological processes, including neuron differentiation and the regulation of cell differentiation **(Figure 2D)**. The results of the KEGG pathway enrichment of differential expression genes were consistent with the GO classification and annotation data. Since most of these results were associated with neurons, we sought to determine the relationship between melatonin and muscle differentiation. A Gene Set Enrichment Analysis (GSEA) plot demonstrated a positive correlation between melatonin expression and the signaling pathways of Wnt/β-catenin and myogenesis **(Figure 2E-F)**.

### GSK3 and β-catenin signals are involved in muscle differentiation

**Figure 3:**
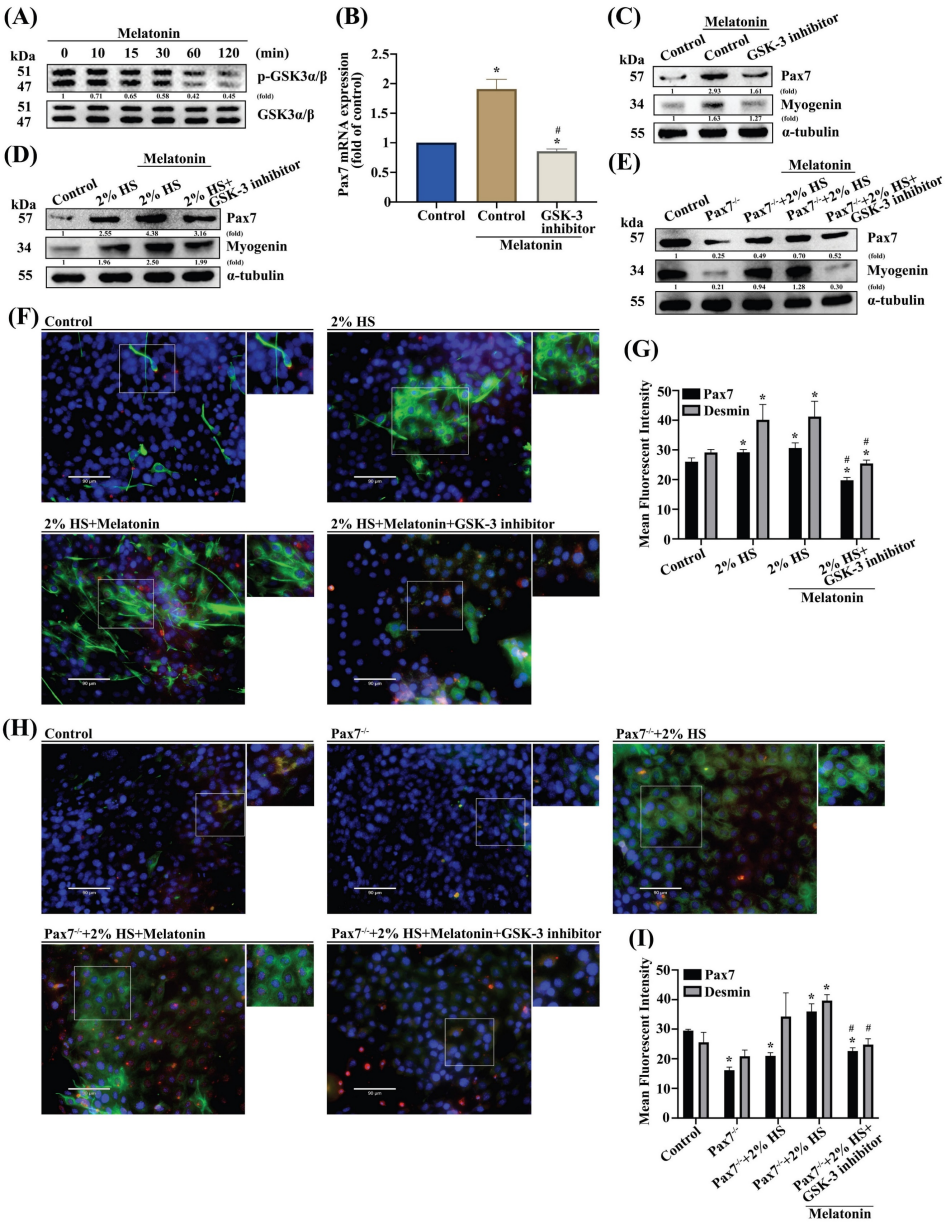
** GSK3β is involved in melatonin-induced upregulation of Pax7 expression. (A)** C2C12 cells were incubated with melatonin (300 μM) for the indicated time intervals and cell lysates were subjected to immunoblotting to detect total protein levels and phosphorylation status of the indicated proteins (N=3). **(B-C)** C2C12 cells were treated with the GSK3β inhibitor for 30 min, then *Pax7* mRNA levels were quantified by qRT-PCR **(B)**, while Pax7 and myogenin protein expression were quantified by immunoblot (N=3)** (C)**. **(D-E)** Myoblasts were incubated with DM for 3 days, then Pax7 and myogenin were examined by Western blot in WT C2C12 and Pax7^-/-^ C2C12 cells (N=3).** (F)** Myoblasts were incubated with DM for 3 days, then examined for Pax7 and desmin markers and **(G)** quantified by immunofluorescent double-staining (N=3).** (H)** WT C2C12 and Pax7^-/-^ C2C12 cells were incubated with DM for 3 days, then treated with the GSK3β inhibitor for 30 min, followed by melatonin treatment before **(I)** quantifying Pax7 and desmin protein expression using immunofluorescent double-staining (N=3). Results are expressed as the means ± SD of at least 3 independent experiments. *p < 0.05 compared with the control group; #*p* < 0.05 compared with the melatonin-treated group.

**Figure 4:**
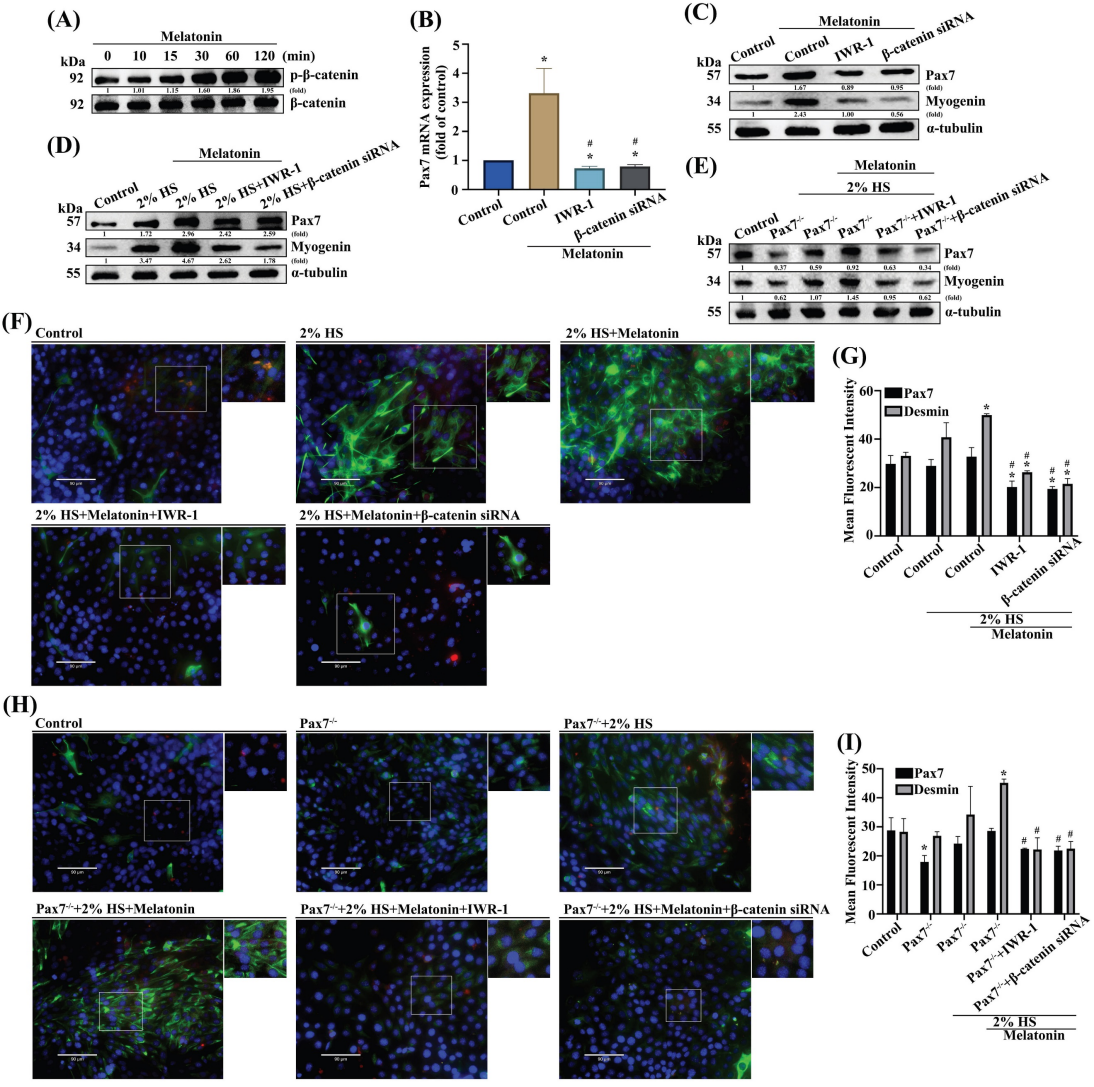
** β-Catenin is downstream of the GSK3β signaling pathway in melatonin-induced increases in Pax7 expression. (A)** C2C12 cells were incubated with melatonin (300 μM) for the indicated time intervals and cell lysates were subjected to immunoblotting to detect total protein levels and phosphorylation status of the indicated proteins (N=3). **(B-C)** C2C12 cells were treated with IWR-1 or transfected with β-catenin siRNA before quantifying *Pax7* mRNA levels with qRT-PCR **(B)** and Pax7 and myogenin protein expression by immunoblot **(C)** (N=3). **(D-E)** Myoblasts were incubated with DM for 3 days, and Pax7 and myogenin were examined by Western blot in WT C2C12 and Pax7^-/-^ C2C12**. (F)** Myoblasts were incubated with DM for 3 days, and Pax7 and desmin were examined and **(G)** quantified by immunofluorescent double-staining (N=3). **(H)** WT C2C12 and Pax7^-/-^ C2C12 cells were incubated with DM for 3 days, then treated with IWR-1 or transfected with β-catenin siRNA, followed by melatonin treatment (N=3). **(I)** Pax7 and desmin protein expression were quantified by immunofluorescent double-staining. Results are expressed as the means ± SD of at least 3 independent experiments. *p < 0.05 compared with the control group; #*p* < 0.05 compared with the melatonin-treated group.

Previous studies have shown that Wnt signals that include both canonical and noncanonical pathways are related to myogenesis and regulation of muscle formation 27, 28. Incubating myoblast cells for 2 h with melatonin time-dependently promoted phosphorylation of glycogen synthase kinase β (GSK3β) signaling **(Figure 3A)**. Pretreating cells with the GSK3β inhibitor inhibited melatonin-induced increases in Pax7 mRNA and protein levels **(Figure 3B-C)**. Myoblasts were incubated with DM for 3 days, and the myogenesis markers Pax7 and myogenin were examined by Western blot in wild-type C2C12 **(Figure 3D)** and Pax7 knockdown C2C12 cells **(Figure 3E)**. Immunofluorescent double-staining results showed that after melatonin treatment, GSK3β signaling was involved in DM cells **(Figure 3F)** as well as in Pax7 knockdown C2C12 cells **(Figure 3H)**; these results are quantified as **Figure 3G and 3I**, respectively. Canonical Wnt signaling is associated with β-catenin-dependent phosphorylation 29 , so we analyzed the time-dependent effects of melatonin treatment on β-catenin phophorylation **(Figure 4A)**. Pretreating wild-type C2C12 cells with GSK3β general inhibitors (IWR-1) or transfecting the cells with β-catenin siRNA inhibited melatonin-induced increases in Pax7 mRNA and protein expression **(Figure 4B-C)**. Myoblasts were incubated with DM for 3 days, and the myogenesis markers Pax7 and myogenin were examined by Western blot in wild-type C2C12 **(Figure 4D)** and Pax7 knockdown C2C12 cells **(Figure 4E).** Following melatonin treatment, immunofluorescent double-staining results revealed the involvement of GSK3β and β-catenin signaling in wild-type C2C12 and DM cells **(Figure 4F)**, as well as in Pax7 knockdown C2C12 cells and DM cells **(Figure 4H)**; these results are quantified as **Figure 4G and 4I**, respectively. The data indicate that after 3 days of differentiation, the Pax7 knockdown C2C12 cell line appears to contribute to the inhibition of Wnt signaling in C2C12 cells, suggesting that melatonin activates myoblast differentiation and Pax7 expression through the Wnt signaling pathways.

### miR-3475-3p is involved in melatonin-induced increases in Pax7 expression

**Figure 5:**
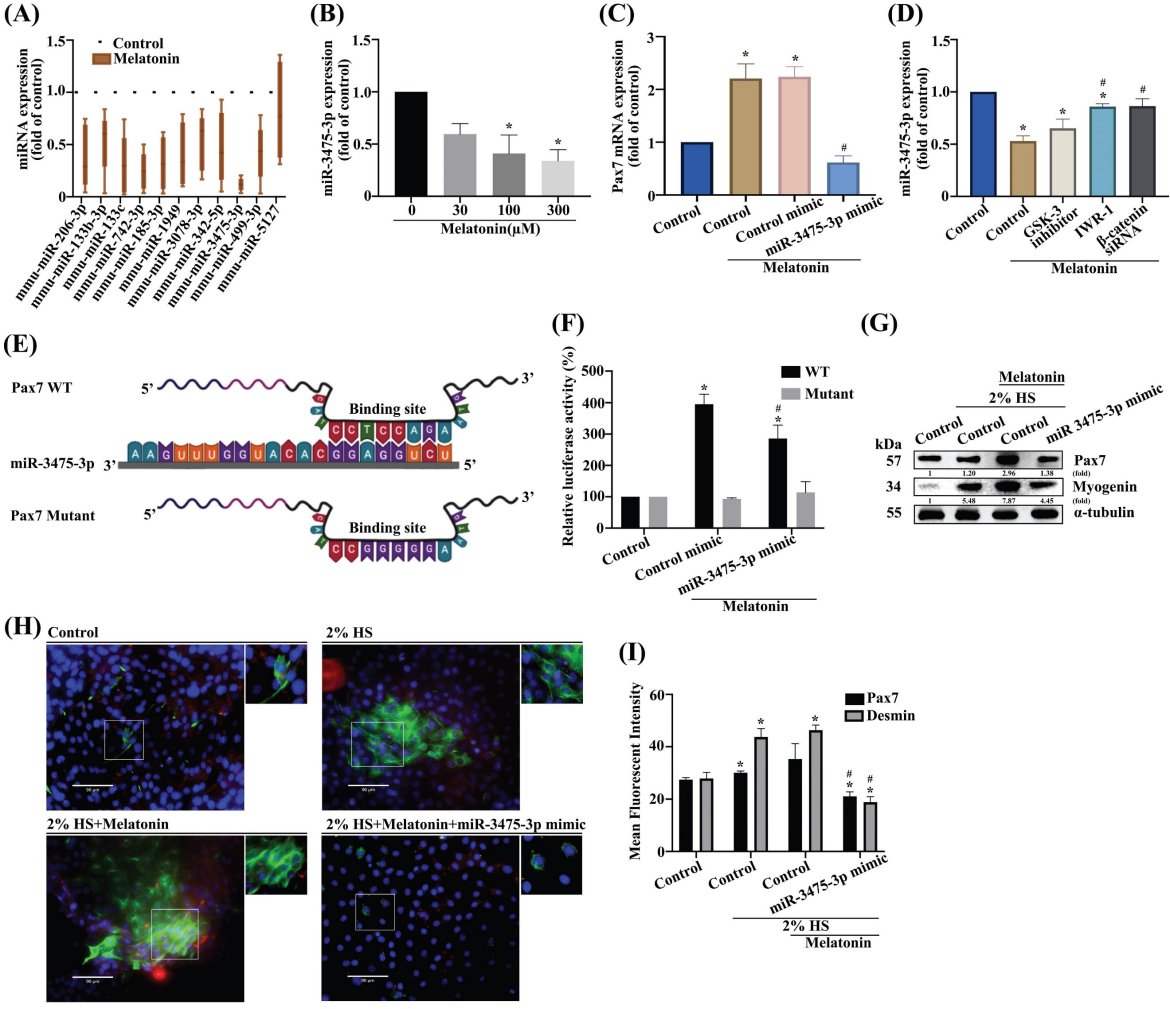
**miR-3475-3p is involved in melatonin-induced increases in Pax7 production. (A)** After analyzing miRNA online databases, 11 candidate miRNAs were examined for *Pax7* mRNA expression using qRT-PCR (N=4). **(B)** Levels of miR-3475-3p expression were measured after different concentrations of melatonin treatment (N=4).** (C)** C2C12 cells were transfected with miR-3475-3p mimic following melatonin treatment and *Pax7* mRNA levels were analyzed by qRT-PCR (N=3).** (D)** WT C2C12 cells were treated with different inhibitors or transfected with β-catenin siRNA, followed by melatonin treatment, then *miR-3475-3p* mRNA levels were analyzed by qRT-PCR. **(E)** The sequence shows a WT Pax7 3′-UTR region containing the miR-3475-3p binding site and a mutant form of this 3′-UTR region. **(F)** C2C12 cells were transfected with WT or mutant luciferase plasmids and luciferase activity was measured by qRT-PCR (N=3). **(G)** Myoblasts were incubated with DM for 3 days, and Pax7 and myogenin were examined by Western blot (N=3).** (H)** Myoblasts were incubated with DM for 3 days, then transfected with miR-3475-3p mimic (N=3). **(I)** Pax7 and desmin markers were quantified by immunofluorescent double-staining. Results are expressed as the means ± SD of at least 3 independent experiments. *p < 0.05 compared with the control group; #*p* < 0.05 compared with the melatonin-treated group.

A number of microRNAs (miRs) act as significant mediators in muscular diseases, including muscle atrophy; their postulated functions relate to muscle differentiation 30-32. To determine which miRNAs target *Pax7*, we screened the online databases miRwalk, miRanda, and Targetscan, for candidate miRs. All 11 identified miRNAs regulated *Pax7* mRNA expression **(Figure 5A)**. Accordingly, we selected miR-3475-3p as a negative regulator for Pax7. Treating myoblasts with melatonin resulted in significant, concentration-dependent decreases in miR-3475-3p levels **(Figure 5B)**. To prove whether miR-3475-3p directly mediates Pax7, we transfected myoblasts with miR-3475-3p mimic and observed a subsequent inhibition of melatonin-induced increases in *Pax7* mRNA levels **(Figure 5C)**. Wild-type C2C12 cells were pretreated with IWR-1 or transfected with β-catenin siRNA prior to melatonin treatment, to ascertain whether melatonin increases Pax7 expression through Wnt signaling pathways; a significant recovery was observed in *miR-3475-3p* mRNA levels **(Figure 5D)**. Next, we constructed a wild-type Pax7 3′-UTR region containing the miR-3475-3p binding site and a mutant form of this 3′-UTR region into the firefly luciferase plasmids **(Figure 5E)**. Melatonin treatment increased the wild-type but not the mutant form of the 3′-UTR firefly luciferase plasmids, which confirmed that miR-3475-3p directly binds to the 3′-UTR of Pax7 **(Figure 5F)**. In addition, myoblasts were transfected with miR-3475-3p mimic and incubated with DM for 3 days. Western blot and immunofluorescent double-staining data for the myogenesis markers Pax7 and myogenin confirmed that miR-3475-3p was involved in melatonin-induced muscle differentiation **(Figure 5G-I)**. These results suggest that miR-3475-3p is likely to be downstream of Wnt signaling during muscle differentiation after melatonin treatment.

### Melatonin improves *in vivo* skeletal muscle dystrophy

**Figure 6:**
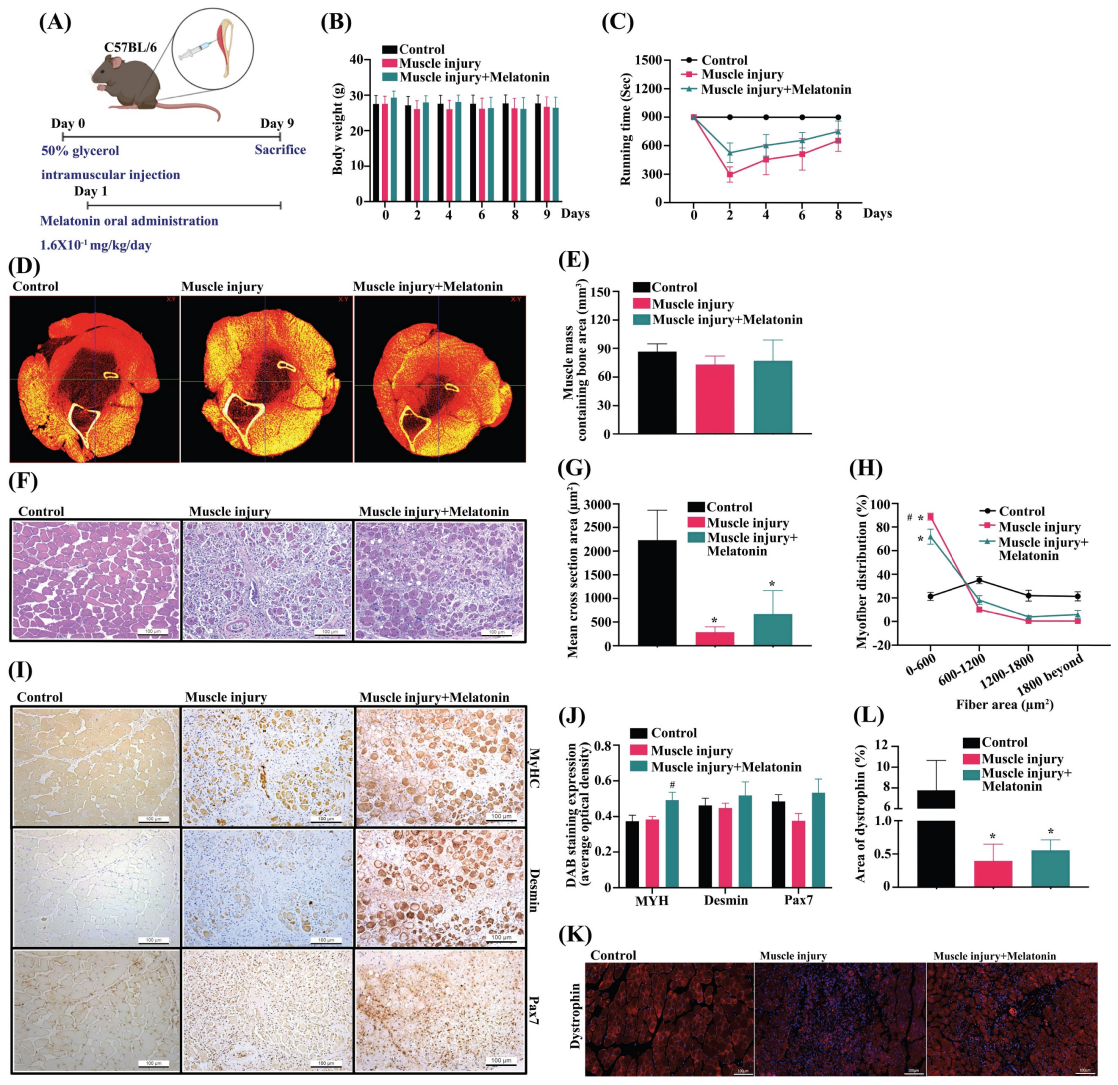
** Melatonin rescues skeletal muscle injury in the glycerol-induced muscle injury mouse model. (A)** The mice were randomly separated into three groups: control; muscle injury; and muscle injury + melatonin oral administration (n=10 mice/group). **(B)** Bodyweight was measured on days 0, 2, 4, 6, 8, and 9. **(C)** Muscle function was evaluated by rotarod testing. **(D)** Results of micro-CT scans of tibialis anterior (TA) muscle after phosphotungstic acid staining (N=4). **(E)** Total volumes of TA muscle and bone were evaluated by micro-CT analysis. **(F)** TA muscles were subjected to hematoxylin and eosin (H&E) staining. Scale bar = 100 µm (N=8). **(G-H)** Cross-sectional areas of TA muscle and myofiber distribution analysis from H&E staining (N=6). **(I)** TA muscle was subjected to immunohistochemical staining and **(J)** MyHC, desmin and Pax7 protein were quantified using densitometric analysis performed by ImageJ software. Scale bar = 100 µm (N=6).** (K)** Dystrophin staining is shown in red coloring; nuclei are marked by DAPI staining in blue coloring. Scale bar = 100 µm (N=6). **(L)** Quantification of TA muscle cross-sectional areas after dystrophin staining. Results are expressed as means ± SD. *p < 0.05 compared with the control group, #*p* < 0.05 compared with the muscle atrophy group.

Intramuscular glycerol injection disrupts rabbit skeletal muscle within the first 24 h of injection, followed by extensive regenerative changes between 7 and 14 days of injection 33. We then investigated the effects of melatonin treatment upon skeletal muscle dystrophy in C57BL/6J mice **(Figure 6A)**. Body weights did not differ significantly among study groups **(Figure 6B)**. Rotarod performance in the glycerol-induced muscle injury group was less accurate than in the control group **(Figure 6C)**. When melatonin treatment was administered to the mice with glycerol-induced muscle injury, rotarod performance was significantly improved **(Figure 6C)**. Micro-CT images of the TA muscle after PTA staining showed that melatonin treatment reversed glycerol-induced reductions in TA muscle thickness and TA muscle mass **(Figure 6D-E)**. Moreover, H&E staining revealed marked hind limb muscle morphology and muscle fiber damage in the muscle injury group compared with the control group **(Figure 6F)**. Analysis of a cross-sectional area of TA muscles and myofiber distribution demonstrated significant improvements in the melatonin treatment group compared with controls **(Figure 6G-H)**. Glycerol-induced muscle injury significantly promoted levels of melatonin and Pax7 protein expression in TA muscle, and decreased muscle-specific differentiation markers MyHC, desmin and Pax7, as indicated by IHC staining **(Figure 6I)**. Previous research has demonstrated that glycerol causes muscle necrosis through the loss of dystrophin protein marker expression in fibers 34. In this study, immunofluorescent data showed that melatonin significantly reversed dystrophin protein expression in muscle fibers **(Figure 6K)**. IHC and dystrophin staining results are quantified as **Figure 6J and 6L,** respectively. These results indicate that melatonin-induced increases in Pax7 expression rapidly rescue skeletal muscle differentiation and improve muscle fiber morphology in glycerol-induced skeletal muscle injury.

## Discussion

Skeletal muscle regeneration improves muscle dystrophy and is characterized by muscle differentiation and myogenesis 15, 35. Several papers have shown that melatonin reduces oxidative stress and inflammation in damaged or diseased skeletal muscle 36-38. However, the effects of melatonin upon muscle dystrophy remain unknown. This study demonstrates that melatonin enhances the biomechanical characteristics of the skeletal muscle differentiation by increasing Pax7 expression. Our *in vitro* results showed significantly myotubes differentiation after 3-days DM incubation. The results of bioinformatics analysis suggest that melatonin participates in skeletal muscle 3-days differentiation and proliferation by regulating the corresponding target genes. During the process of myoblast differentiation into myocytes, Pax7 functions as a molecular switch that impairs skeletal muscle regeneration after acute muscle injury 39. Although a previous study reported that Pax7 plays an important role in muscle cell differentiation 40, the mechanism that regulates Pax7 differentiation during muscle dystrophy needs to be fully defined. Previous study indicated that MuRF-2 was higher than MuRF-1 in atrophic mice 41. Thus, our results showed skeletal muscle atrophy markers with atrogin-1, MuRF-2, and myostatin. In our study, melatonin increased the synthesis of myogenic markers (IGF-1, Pax7, MyoD), decreased the expression of skeletal muscle atrophy markers (Atrogin-1, MuRF-2 and myostatin), and enhanced the expression of myotube-specific differentiation markers (MyHC and myogenin). Since the efficiency of the Pax7 knockdown was approximately decreased in 46%, therefore, the protein production of Pax7 was still upregulated by the DM and melatonin in C2C12. Our study evidence supports the contention that a balance of these markers is essential for maintaining myogenesis, suggesting that melatonin regulates muscle dystrophy via these myogenic markers. We describe a novel mechanism whereby melatonin contributes to myoblast differentiation by regulating Pax7 expression.

Several pathways play important roles in the regulation of myogenesis, whereby myogenic progenitor cells activate muscle differentiation and MyoD expression during muscle injury 42. The targets of Wnt/β-catenin that drive muscle differentiation are of considerable interest. Our *in vitro* results show that Pax7 is downstream of Wnt signaling during myoblast differentiation. It is known that miRNAs participate in multiple regulatory pathways in skeletal muscle 43. An *in vivo* miRNA report has shown that inhibition of miR-29b attenuated immobilization-induced muscle atrophy 44. However, we only investigated the *in vitro* role of miR-3475-3p, so further research is needed to investigate the *in vivo* role of miR-3475-3p in muscle atrophy in response to different atrophic stimuli in animal models. The* in vitro* results suggest that melatonin induces myoblastic differentiation and Pax7 expression through the Wnt signaling pathway. We have also shown for the first time that miR-3475-3p is negatively regulated by melatonin in myoblasts in a Pax7-dependent manner.

Skeletal muscle is characterized by regeneration in response to different types of injuries or myopathy 45, 46. Previous studies have demonstrated that glycerol induces injury by adipocyte infiltration or deposition of collagen fibers 8, 47. Our *in vivo* results demonstrate early-stage muscle dystrophy following rapid induction of muscle injury induced by glycerol treatment. IHC staining results showed that glycerol-induced muscle dystrophy significantly promoted levels of melatonin and Pax7 protein expression in TA muscle, and decreased muscle-specific differentiation markers MyHC, desmin, and Pax7 on day 7 during the early stage of muscle injury in mice. Notably, evidence from other research has shown that glycerol-induced muscle regeneration occurs during the late stage of muscle atrophy, at 14 days after glycerol injection 48. In our model of glycerol-induced muscle injury, rotarod and micro-CT results reveal significant muscle recovery in mice treated with oral melatonin compared with the untreated group of mice. These data suggest that melatonin may effectively rescue glycerol-induced muscle dystrophy in the early stage of injury. In addition, the role of melatonin in other muscle atrophy models, including cardiotoxin-induced muscle atrophy, aging mice and pathologic sarcopenic mice, should be further examined in future work.

**Figure 7:**
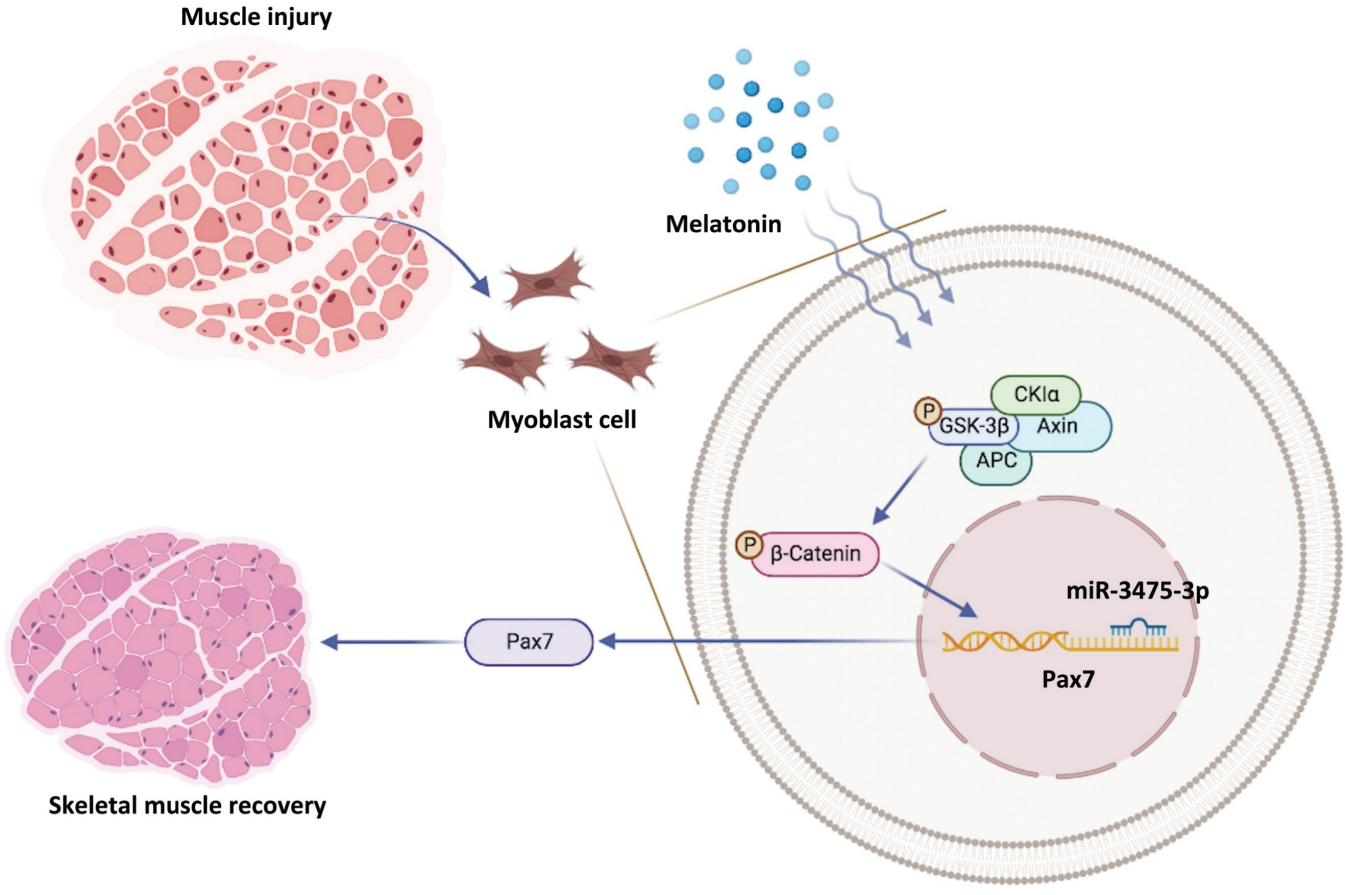
Schematic diagram illustrating the *in vitro* and *in vivo* effects of the melatonin/Pax7 axis upon skeletal muscle injury.

In conclusion, we have demonstrated for the first time that melatonin-induced increases in Pax7 expression lead to skeletal muscle differentiation in vitro, which quickly rescues glycerol-induced muscle injury *in vivo* (Figure 7). Our data suggest that the melatonin/Pax7 axis could serve as an important therapeutic target in muscle differentiation.

## Supplementary Material

Supplementary figure and materials.

## Abbreviations

MuRF-1: muscle RING-finger-1
MyoD: myogenic differentiation
Pax7: paired box 7
IGF-1: insulin-like growth factor 1
MyHC: myosin heavy-chain
siRNA: small-interfering RNA
DMEM: Dulbecco's Modified Eagle's Medium
DM: differentiated medium
FPKM: fragments per kilobase per million
qRT-PCR: Quantitative real-time polymerase chain reaction
DAPI: 4,6-Diamidino-2-phenylindole
TA: tibialis anterior
rpm: revolutions per minute
micro-CT: micro-computed tomography
GPU: graphics processing unit
H&E: hematoxylin and eosin
GO: Gene Ontology
GSEA: Gene Set Enrichment Analysis
GSK3β: glycogen synthase kinase β
IWR-1: GSK3β general inhibitors
miR: microRNA
IHC: Immunohistochemistry
SD: standard deviation
Myog: myogenin
KEGG: Kyoto Encyclopedia of Genes and Genomes
