# Supplementary material for: Melatonin improves muscle injury and differentiation by increasing Pax7 expression
Source: Int J Biol Sci. 2023 Jan 22;19(4):1049–62. doi: 10.7150/ijbs.79169 (PMC10008686; doi:10.7150/ijbs.79169)
Supplement: Supplementary file 1 — Supplementary figure and materials. [file ijbsv19p1049s1.pdf]

Supplementary Figures

Supplementary Figure S1.

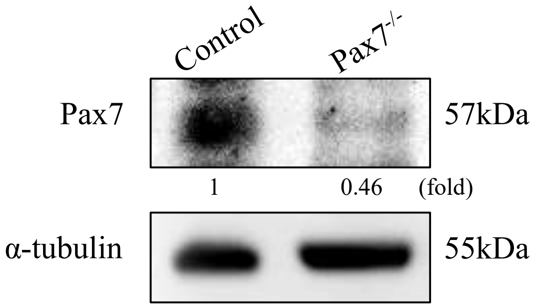

Supplementary Figure S1. Wild-type (WT) C2C12 cells and Pax7 knockdown C2C12 ($Pax7^{-/-}$) cells were examined by Western blot.

Supplementary Materials

Antibodies

Protein	Application	Vendor	Catalog number
α-tubulin	WB	Abcam	ab176560
β-catenin	WB	Santa Cruz	sc-133240
Desmin	WB, IHC, IF	Abcam	ab15200
Dystrophin	WB, IF	Abcam	ab15277
GSK-3α/β	WB	Santa Cruz	sc-56913
Myogenin	WB	Santa Cruz	sc-52903
MyHC	WB, IHC	ThermoFisher	PA5-31466
Pax7	WB, IHC, IF	Novus Biologicals	NBP2-32894
p-β-catenin	WB	Cell signaling	2009S
p-GSK-3β	WB	Santa Cruz	sc-135653

Primers

Gene	Species	Forward primer	Reverse primer
GAPDH	Mouse	ACCACAGTCCATGCCATCAC	TCCACCACCCTGTTGCTGTA
Atrogin 1	Mouse	GAGTGGCATCGCCCAAAAGA	TCTGGAGAAGTTCCCGTATAAGT
IGF-1	Mouse	GGACCGAGGGGCTTTTACTT	GTGGGGCACAGTACATCTCC
MyoD	Mouse	GAGGATCCGATGGAGCTTCTATCG	CGGATCCTCTCAAAGCACCTGATA
Myostatin	Mouse	CTGGTCCTGGGAAGGTTACA	ACGCTACCACGGAAACAATC
Murf 2	Mouse	GTCCTGGTGACACAGATTGGAT	TGCTGCCTATGTGCTTCTCA
Pax7	Mouse	GGTCCCCAGGATGATGAGA	TTGATGAAGACCCCACCAAG

Inhibitors

Gene	Name	Vendor	Catalog number
GSK-3β	GSK-3 inhibitor	Santa Cruz	sc-202634A
Wnt/β-catenin	IWR-1	Enzo	BML-WN103

Treatment

Drug	Vendor	Catalog number
Melatonin	Sigma	SLBQ9501V

shRNA

Gene	Species	Vendor	Sequence
Pax7	Mouse	RNAi core, Taiwan	CCGTCACAAGATAGTGGAAAT

siRNA

Gene	Species	Vendor	Catalog number
β-catenin (siRNA)	Mouse	Santa Cruz	sc-29210

Mimics

Mimic	Species	Vendor	Sequence (5'-3')
miR-3475-3p	Mouse	AllBio	UCUGGAGGCACAUGGUUUGAA
N.C.	Mouse	AllBio	UUGUACUACACAAAAGUACUG

3' UTR of Pax7

Gene	Species	Vendor	Sequence
Pax7 miR-3475-3p WT	Mouse	MDBio, Inc.	CCTCCAGA
Pax7 miR-3475-3p MUT	Mouse	MDBio, Inc.	CCGGGGGA
